# Primary squamous cell carcinoma of the pleura treated with nivolumab

**DOI:** 10.1002/rcr2.516

**Published:** 2020-02-05

**Authors:** Ioanna Sigala, Nektarios Alevizopoulos, Konstantinos Elefteriou, Niki Gianniou, Ioannis Kalomenidis

**Affiliations:** ^1^ 1st Department of Critical Care and Pulmonary Medicine Evaggelismos Hospital, National and Kapodistrian University of Athens Athens Greece; ^2^ Department of Oncology Evaggelismos Hospital Athens Greece

**Keywords:** Immunotherapy, nivolumab, primary squamous cell carcinoma of the pleura

## Abstract

Malignant pleural effusions usually manifest in the course of metastatic cancer disease. Primary pleural tumours are rare with mesothelioma being by far the most common. Primary squamous cell carcinoma of the pleura (PSCCP) is extremely rare. It is usually asymptomatic, until it invades the pleura presenting pain as the first symptom. Our knowledge about its treatment or prognosis is limited due to its rarity. We present the case of a 48‐year‐old man who presented with persistent right‐sided thoracic pain with chest computed tomography (CT) scan demonstrating a right‐sided pleural effusion and pleural mass invading the ribs. Ultrasound‐guided biopsy revealed a PSCCP. Positron emission tomography staging demonstrated metastatic lung and lymph node involvement precluding surgical therapy. We provide information about treatment, including immunotherapy as well as extended follow‐up course. Immunotherapy with nivolumab resulted in prolongation of survival with good quality of life.

## Graphical abstract

We report the case of a 48‐year‐old man who presented with persistent right‐sided thoracic pain with chest computed tomography scan demonstrating a right‐sided pleural effusion and pleural mass invading the ribs. Ultrasound‐guided biopsy revealed a primary squamous cell carcinoma of the pleura. Positron emission tomography staging demonstrated metastatic lung and lymph node involvement precluding surgical therapy. Immunotherapy with nivolumab resulted in prolongation of survival with good quality of life.

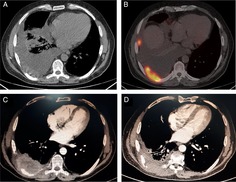

## Introduction

Malignant pleural effusions are common and usually present metastatic involvement of the pleura during the course of neoplasms such as lung or breast cancer. On the contrary, primary pleural tumours are rare with mesothelioma either diffuse or localized being by far the most common. Primary squamous cell carcinoma of the pleura (PSCCP) is extremely rare with only case reports published in the literature. It is usually asymptomatic at the beginning until it invades the surrounding structures presenting pain as a symptom. Its course is to progress locally and metastasize. Our knowledge is limited regarding the treatment and long‐term prognosis of PSCCP.

## Case Report

**Figure 1:**
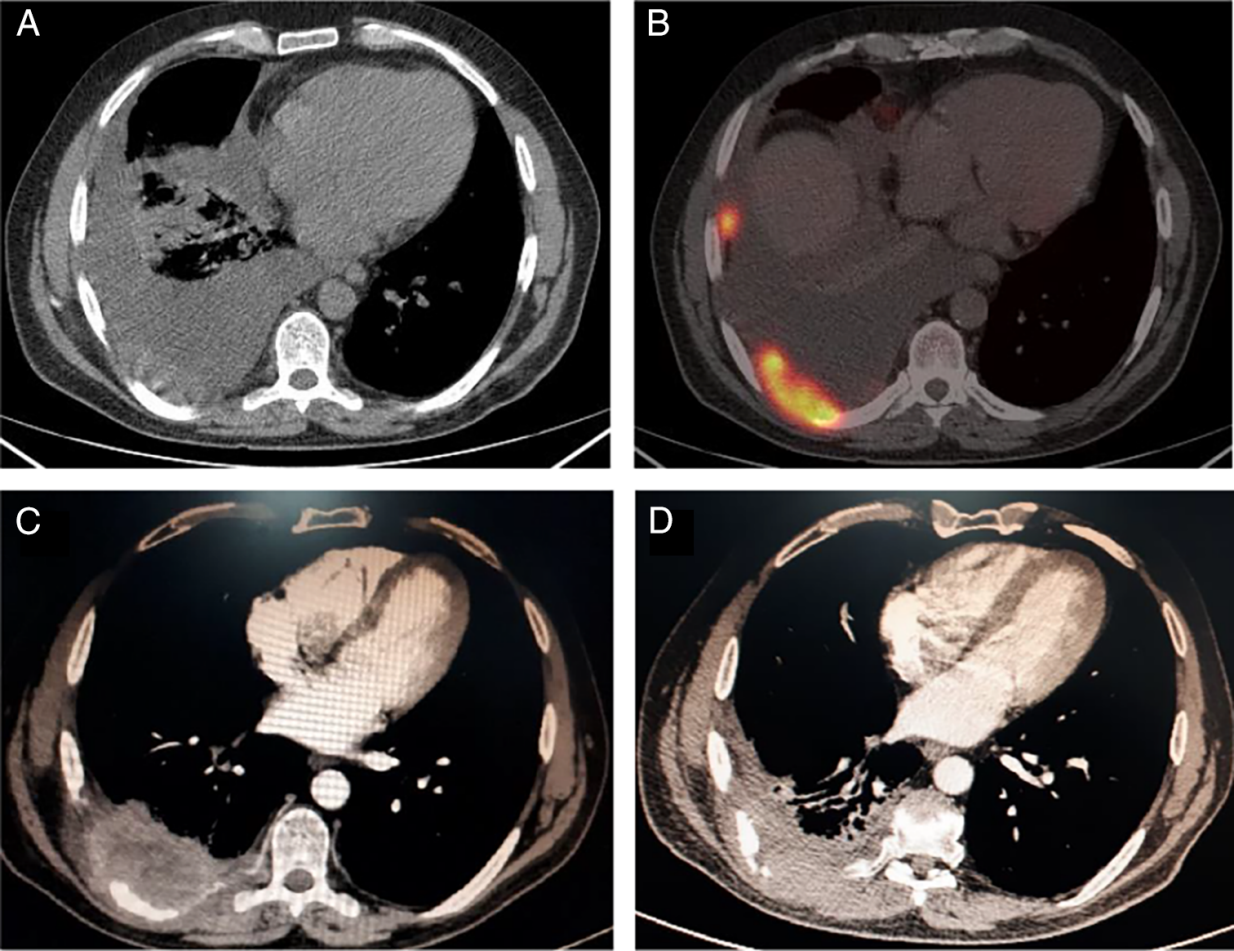
Computed tomography (CT) scan sequential imaging of primary squamous cell carcinoma of the pleura. (A) CT and (B) positron emission tomography (PET) CT images at the time of diagnosis depicting the pleural and one of the pleural nodules. (C) Image at the completion of first‐line chemotherapy—six months from initial diagnosis—showing tumour growth with necrosis and rib invasion. (D) Image 19 months after initial diagnosis (13 months of treatment with nivolumab) showing tumour stability.

A 48‐year‐old man, active smoker, presented with persistent right‐sided thoracic pain lasting more than a month. Chest computed tomography (CT) demonstrated a right‐sided pleural effusion and a 6.4‐cm pleural mass at the level of the right lower lobe invading the eighth and ninth ribs (Fig. 1A). Smaller nodules all over the pleura were also found. Ultrasound‐guided biopsy revealed a PSCCP (p63+, CK5/6+, p40+, thyroid transcription factor (TTF‐1)−, wild‐type epidermal growth factor receptor (EGFR), and <1% programmed cell death‐ligand 1 (PD‐L1) receptors positivity). Positron emission tomography scan demonstrated abnormal uptake at the right‐sided pleural mass and nodules [maximum standardized uptake value (SUVmax) 32] (Fig. 1B), at two pulmonary nodules in the left lung (SUVmax 4.5), at the right epiphrenic, and at the subcarinal lymph nodes (SUVmax 8.7).

**Figure 2:**
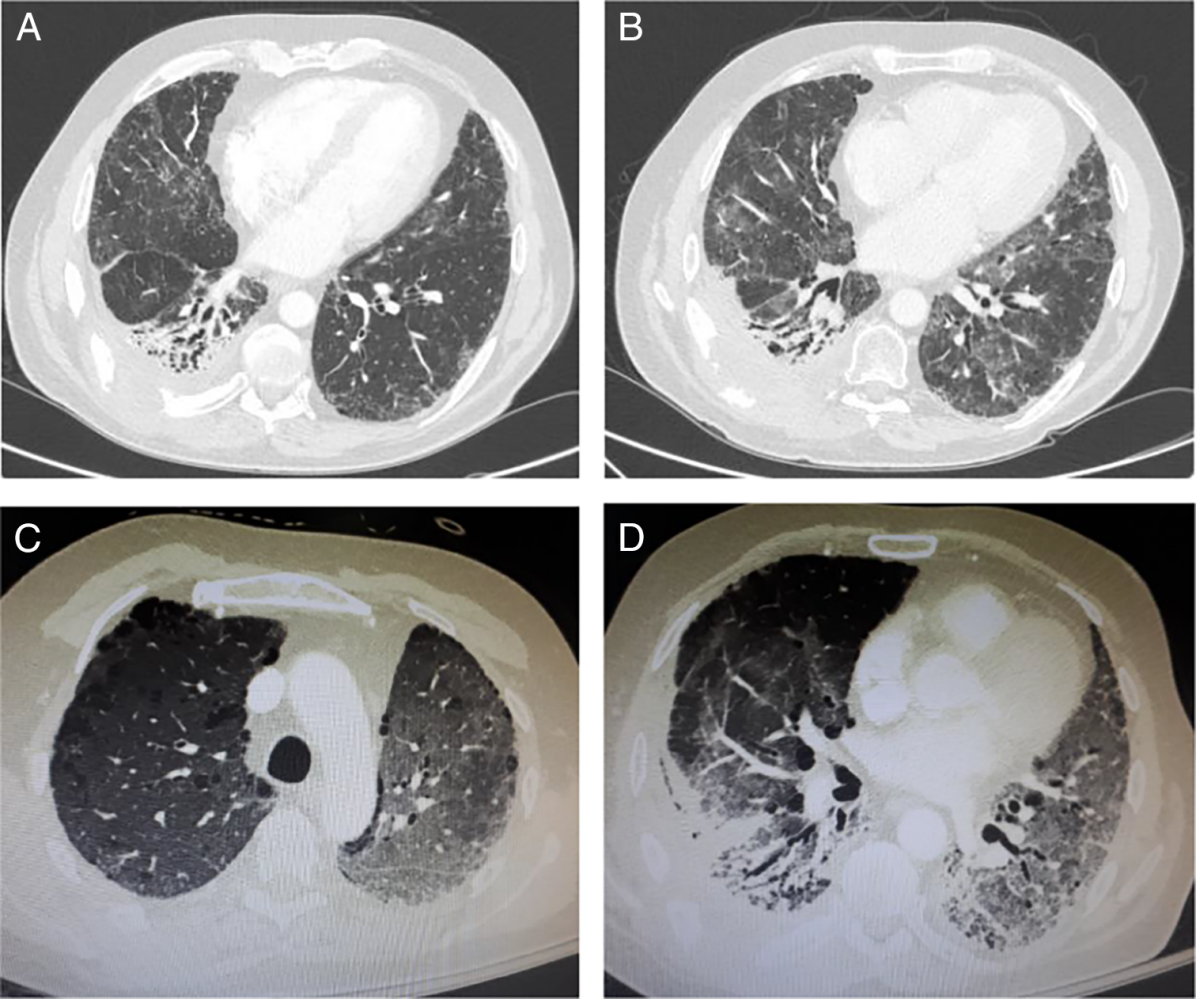
Computed tomography (CT) scan sequential imaging of primary squamous cell carcinoma of the pleura. (A, B) Evidence of pulmonary fibrosis on the right lower lobe due to radiation treatment, (A) one month and (B) seven months after radiation treatment. (C, D) Ground‐glass opacities all over the lung—lung toxicity due to nivolumab treatment.

The patient received six cycles of platinum‐based combination with taxane (classic cis‐platinol and docetaxel), with good initial response (resolution of pleural effusion and symptomatic improvement). No side effects associated with chemotherapy were documented. However, repeat chest CT scan at the completion of treatment (six months from diagnosis) demonstrated disease progression (Fig. 1C). The patient switched to immunomodulation treatment with nivolumab (programmed cell death‐1 (PD‐1) inhibitor) with complementary local radiation therapy. Nivolumab was administered at a dose of 3 mg/kg, with a total infusion dose of 240 mg/15 days delivered. Radiotherapy was chosen on a palliative basis to control local extension of the tumour; 50 Gy was stereotactically applied and rapid pain relief was observed. The disease remained stable for 13 months with nivolumab treatment (Fig. 1D), with excellent quality of life and no side effects apart from the radiological evidence of local pulmonary fibrosis at the site of radiation (Fig. 2A, B). Twenty months after the initial diagnosis, our patient presented with a solitary brain metastasis that was treated with Cyberknife radiation. After this point, tumour behaviour changed, exhibiting fast local growth despite nivolumab treatment.

**Figure 3:**
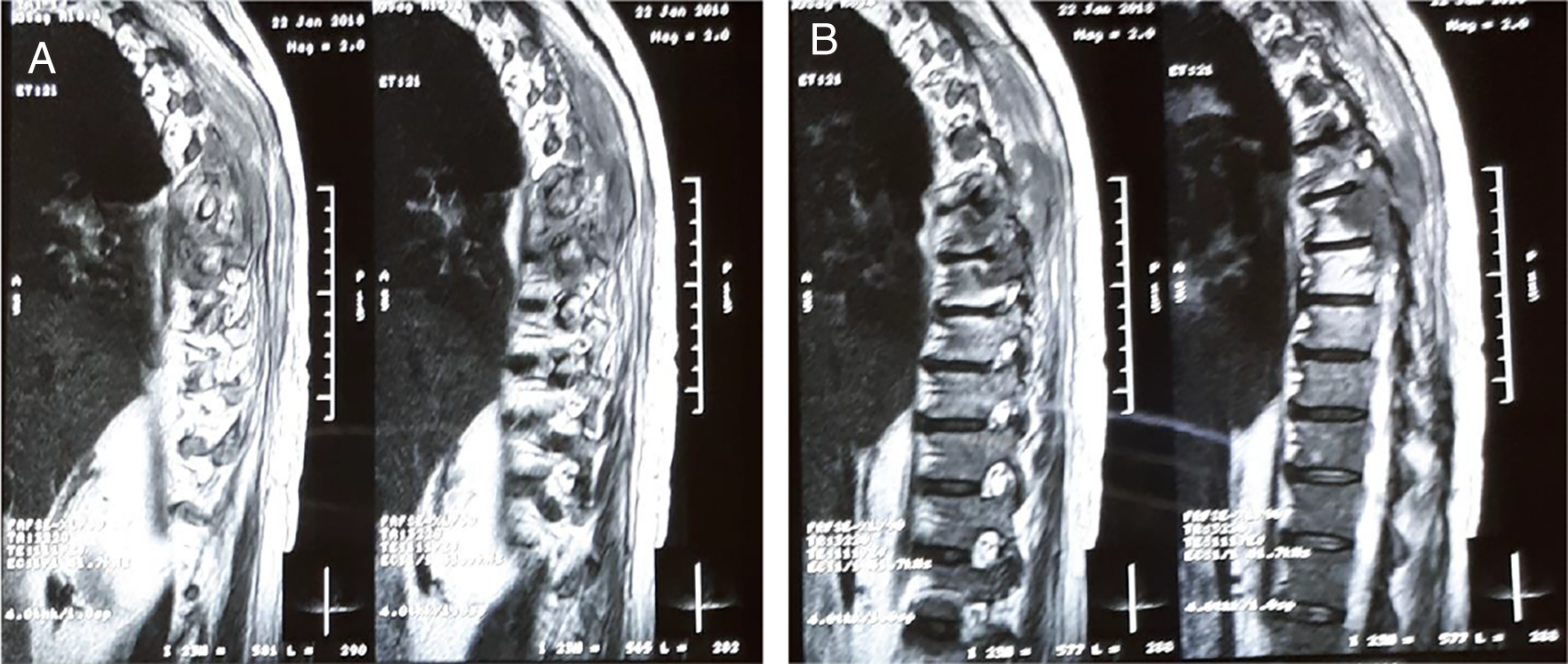
Magnetic resonance imaging (MRI) imaging of thoracic vertebra depicting tumour invasion on T6–T8 vertebra and into the root canal with resulting pressure into the spinal cord.

Our patient's clinical course was complicated with pneumonitis due to nivolumab toxicity (Fig. 2C, D) resulting in severe respiratory failure (21 months from diagnosis—14th month of nivolumab treatment). He received 1 mg/kg prednisolone for Grade III pneumonitis with good response, tapered over four weeks, and followed by permanent discontinuation of nivolumab. Furthermore, one month later, he developed neurological symptoms (lower limb paralysis, urinary retention, and faecal incontinence) and magnetic resonance imaging of the spine revealed local invasion of the tumour to the T6–T8 vertebra and into the root canal with resulting pressure into the spinal cord (Fig. 3). A palliative operation for cord decompression was performed, resulting in significant neurological improvement. At this point, our patient's performance status was obviously compromised and the decision for comfort care was made. He passed away three months later, 24 months after the initial diagnosis.

## Discussion

PSCCP is an extremely rare tumour. Its cause is not known although earlier reports connected it with chronic inflammation or surgery 1, 2, 3. PSCCP is a tumour that invades locally and metastases. It remains asymptomatic until the invasion of surrounding tissues cause persistent pain as the first main symptom. It usually has to be differentiated from malignant mesothelioma. In that case, immunohistochemistry with nuclear staining for P63 and P40 is useful as they both are positive in PSCCP but not in mesothelioma 4.

Anti‐tumour immunotherapy with immune check point inhibitors especially targeting PD‐1 receptor or its PD‐L1 has changed the landscape in the treatment of advanced non‐small cell lung cancer (NSCLC) exhibiting improved survival and response rates combined with a less toxic profile 5. Nivolumab is a human IgG4 monoclonal antibody that inhibits PD‐1 activity. Apart from NSCLC, it is indicated for melanoma, renal cell carcinoma, head and neck cancer, and Hodgkin lymphoma. A number of adverse effects such as skin rash, pneumonitis, colitis, hepatitis, endocrinopathies (thyroid gland and hypophysitis), and rheumatological are related to immune check point inhibitor therapy and consist of an immunological reaction to the activation of lymphocytes (immune‐related adverse events). Pneumonitis is more common in patients with NSCLC and in patients treated with combination of inhibitors for both PD‐1 and cytotoxic T‐lymphocyte‐associated antigen‐4 (CTL4).

We present here the case of a patient with metastatic PSCCP, with no history of pleural inflammation or operation. The immunohistochemistry profile of the patient was suggestive for PSCC (p63+, CK5/6+, p40+, and TTF‐1−). The combination of p40+ and TTF‐1− was highly indicative of the de novo genesis of SCCP as lung parenchyma was free of disease and no special immunochemistry markers could stain the cancer arising from lung. Moreover, positivity for both p63 and p40 differentiated it from mesothelioma as mentioned above. Apart from surgical removal, evidence on the role of systemic treatment in PSCCP is limited. To our knowledge, immunotherapy has not been previously administered in patients with PSCCP. The decision to use nivolumab was based on the established algorithm that, nowadays, squamous‐cell lung cancers responded excellently to second‐line therapy with immunotherapies molecules. The patient was informed and consented to the use of this treatment as first‐line platinum‐based therapy has failed. Immunotherapy resulted in extremely prolonged disease control.

### Acknowledgement

We thank National and Kapodistrian University of Athens for funding the publication of the paper.

### Disclosure Statement

Appropriate written informed consent was obtained for publication of this case report and accompanying images.
